# Performance of matrix-assisted laser desorption/ionization time-of-flight mass spectrometry (MALDI-TOF-MS) in diagnosis of ovarian cancer: a systematic review and meta-analysis

**DOI:** 10.1186/s13048-019-0605-2

**Published:** 2020-01-10

**Authors:** Kexin Li, Yuqing Pei, Yue Wu, Yi Guo, Wei Cui

**Affiliations:** State Key Laboratory of Molecular Oncology, Department of Clinical Laboratory, National Cancer Center/National Clinical Research Center for Cancer/Cancer Hospital, Chinese Academy of Medical Sciences and Peking Union Medical College, Beijing, 100021 China

## Abstract

**Background:**

To evaluate the diagnostic performance of matrix-assisted laser desorption/ionization time-of-flight mass spectrometry (MALDI-TOF-MS) for ovarian cancer.

**Patients and methods:**

A thorough research was conducted in PubMed, Web of Science and Embase (until November 2018) to identify studies evaluating the accuracy of MALDI-TOF-MS for ovarian cancer. Using Meta-Disc1.4, Review Manager 5.3 and Stata 15.1 software to analyze the pooled results: sensitivity, specificity, positive likelihood ratio (PLR), negative likelihood ratio (NLR), diagnostic odds ratio (DOR), and 95% confidence intervals (CI). The summary receiver operating characteristic curves (SROC) and area under the curve (AUC) show the overall performance of MALDI-TOF-MS.

**Results:**

Eighteen studies were included in the meta-analysis. Methodological quality analysis of the included studies showed that these articles were at low risk of bias and applicability concerns in total. Summary estimates of the diagnostic parameters were as follows: sensitivity, 0.77 (95% CI: 0.73–0.80); specificity, 0.72 (95% CI: 0.70–0.74), PLR, 2.80 (95% CI: 2.41–3.24); NLR, 0.30 (95% CI: 0.22–0.40) and DOR, 10.71 (95% CI: 7.81–14.68). And the AUC was 0.8336. Egger’s test showed no significant publication bias in this meta-analysis.

**Conclusion:**

In conclusion, MALDI-TOF-MS shows a good ability for diagnosing ovarian cancer. Further evaluation and optimization of standardized procedures are necessary for complete relying on MALDI-TOF-MS to diagnose ovarian cancer.

## Introduction

Ovarian cancer (OC) is one of the leading causes of cancer-related death among the gynecological malignancies in women [1]. High malignant degree and heterogeneity, occult location and vague clinical symptoms in the early stage are the chief clinical characteristics [2]. Moreover, metastases have occurred before clinical symptoms in most ovarian cancer patients. Thus, it’s not until advanced that ovarian cancer has been diagnosed. 70% of diagnosed patients are at FIGO stages III and IV, in which the 5-year survival rate is under 30%. Fortunately, if it is detected earlier, the survival rate will reach into 90% at FIGO stage I [3]. Hence, early diagnosis is highly significant to improve prognosis and increase the survival rates.

Nowadays, the common clinical diagnostic biomarkers of OC include cancer antigen 125(CA125), human epididymis protein4 (HE4) and carcinoembryonic antigen (CEA). The sensitivity of CA125 in early ovarian cancer is less than 50%, and HE4 combined with CA125 can discriminate cases of endometriosis cyst and ovarian cancer [4]. CEA is a broad-spectrum tumor marker that can increase sensitivity and specificity in combination with CA125 and HE4 in postmenopausal women [5]. But the sensitivity and specificity cannot meet the clinical demand. In addition, the expression of tumor markers in different historical types and disease stages may change. For this reason, researchers focus on the identification of blood markers that express at the different stages, multiple tumor markers are needed to improve accuracy. Therefore, it is necessary to develop sufficient diagnostic markers and methods.

Recently, the development of proteomics and detection equipment and technology of MS are increasing rapidly, the number of studies of proteins and peptides also increases gradually. Matrix-assisted laser desorption/ionization time-of-flight mass spectrometry (MALDI-TOF-MS), as a new mass spectrometry technology, can successfully detect multiple biomarkers related to diseases, especially in the aspects of diagnosis and treatment of tumor [6–8]. It also can quickly detect some specific phase protein expression in the blood and other body fluid and assist in establishing therapeutic schedule, predicting the outcome of treated patients, and estimating the possibility of recurrence [9]. By this technology, many studies showed that detecting the multiple peptides can improve the diagnosis and prognosis in colorectal cancer, breast cancer, gastric cancer and prostate cancer [10–12].

Now, several studies that have reported the diagnostic value of this method for ovarian cancer show controversial results. So, we propose to evaluate the diagnostic performance of MALDI-TOF-MS for ovarian cancer through meta-analysis.

## Materials and methods

### Search strategy and inclusion /exclusion criteria

We conducted a search in PubMed, Web of Science and Embase (until November 2018) to identify studies that evaluate the diagnostic accuracy of MALDI-TOF-MS for ovarian cancer. The search terms included “MALDI-TOF-MS” or “Matrix-Assisted Laser Desorption-Ionization Mass Spectrometry” or “Laser Desorption-Ionization Mass Spectrometry, Matrix-Assisted” or “Mass Spectrometry, Matrix-Assisted Laser Desorption-Ionization” and “ovarian cancer” or “ovarian neoplasm” or “ovary cancer” or “cancer of the ovary”. We manually searched the reference lists of retrieved articles to identify trials.

All studies were included if they met the following inclusion criteria: (1) Studies of people were diagnosed with histopathology; (2) The measurement of distinguishing ovarian cancer from benign or healthy controls is MALDI-TOF-MS; (3) The sufficient data to calculate the diagnostic parameters (sensitivity, specificity, positive likelihood ratio, negative likelihood ratio) or allow construction the 2 × 2 tables. Animals or cell studies and duplicated studies were excluded.

### Data extraction and quality assessment

Data extraction was performed independently by two reviewers from eligible studies according to the inclusion and exclusion criteria. The third reviewer resolved the disagreements through a discussion when the two authors had differences of opinion. Data collection from each study were extracted: first author; region; year of publication; the number of subjects; age of subjects; specimen type; screening method; statistical software or statistical algorithm; and the number of true positive, false positive, false negative and true negative. QUDAS-2 (Quality Assessment of Diagnostic Accuracy Studies-2) was used to assess the quality of included studies [13]. The quality assessment of studies is divided into two parts: risk of bias and applicability concerns.

### Statistical analysis

Two authors used standard methods recommended for meta-analysis of the diagnostic studies independently. We used Meta-Disc1.4, Review Manager 5.3 and Stata 15.1 software to analyse the result: sensitivity, specificity, positive likelihood ratio (PLR), negative likelihood ratio (NLR), diagnostic odds ratio (DOR), and 95% confidence intervals (CI). The summary receiver operating characteristic curves (SROC) and area under the curve (AUC) show the overall performance of the test. The Spearman correlation coefficient is used to analyse and calculate the threshold effects or non-threshold effects. Also, by plotting the receiver operating characteristics (ROC) curve, the result that observed as a “shoulder-arm” distribution indicated no threshold effects. The heterogeneity of this study was calculated by inconsistency index *I*^*2*^: if *I*^*2*^ > 50%, it turned out to be significant heterogeneity in the study, then we used the random-effects model to calculate the combined statistics; otherwise, a fixed-effect model was implemented. When the heterogeneity effect of this study was present, we performed meta-regression to analyse the source of heterogeneity and conducted the subgroup analysis. Egger’s test was used to analyse the publication bias [14].

## Results

### Included studies and characteristics

**Fig. 1:**
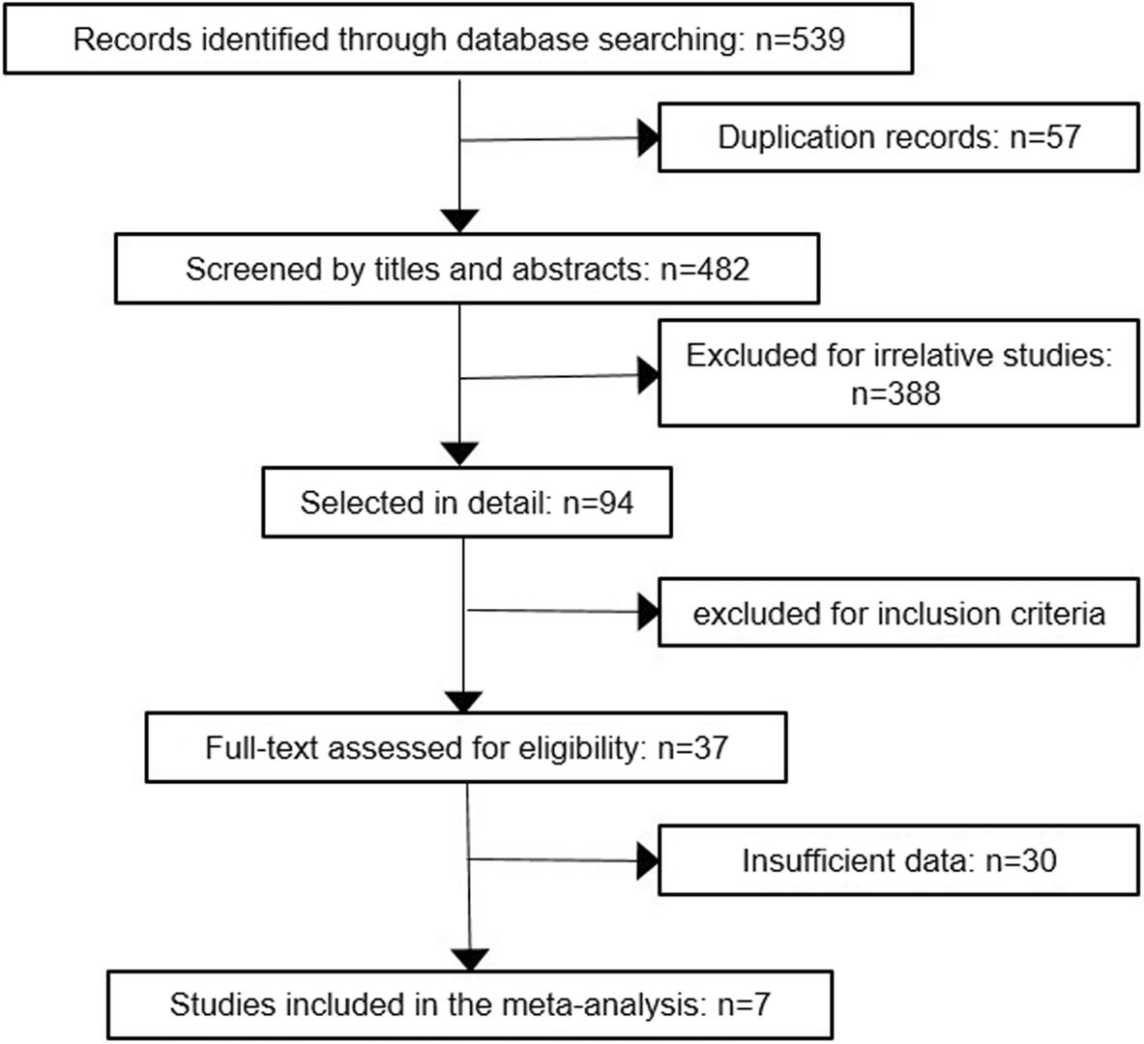
Flow diagram of study selection

Seven articles were included in the meta-analysis [15–21], according to the inclusion and exclusion criteria. The flowchart summarized the process of selection (Fig. 1). From these articles, we extracted the data from eighteen studies, including 592 samples (224 cases and 358 controls). The specimen included serum and plasma. Control group consisted of health or benign ovarian disease patients. Otherwise, Swiatly et al. used the patients with hysterectomy with bilateral salpingo-oophorectomy as the control. The number of subjects ranged from 20 to 188. And the histopathological type was epithelial ovarian cancer. Among these, Zheng et al. used high-grade-serious ovarian cancer and the patients of ovarian cancer in Ali et al. were selected with CA125 > 30-U/ml. In all eligible studies, MALDI-TOF-MS was used to distinguish the patients from the people by measuring the expression of proteins or peptides. These studies also performed the extraction of low abundant proteins or peptides through MALDI-TOF-MS coupled with magnetic beads, such as magnetic beads based weak cation exchange (MB-WXC), C18 Zip Tip, Hydrophobic interaction chromatography magnetic beads C8 (MB-HIC C8) and Cu-immobilized metal affinity chromatography magnetic beads (IMAC-Cu MBs). The characteristics of these studies and patients are listed in Table 1 in detail.

**Table 1:** Characteristics of included studies in the meta-analysis

	Author	Year	Region	Age	Sample type	case/control(n=)	Control type	Histological type	Screening method	TP	FP	FN	TN
1	Zheng, Z.G.	2014	China	55	Serum	34/36	Benign	High-grade-serous ovarian cancer	MB-WCX	18	7	16	29
2	Wu, S	2012	China	48.4	Serum	25/30	Health and benign	EOC	MB-WCX	22	5	3	25
3	Swiatly (a)	2017	Poland	65	Serum	11/12	Bilateral salpingo-oophorectomy	OC	C18 ZipTip	8	4	3	8
4	Swiatly (b)	2017	Poland	65	Serum	11/12	Bilateral salpingo-oophorectomy	OC	C18 ZipTip	9	6	2	6
5	Swiatly (c)	2017	Poland	65	Serum	11/12	Bilateral salpingo-oophorectomy	OC	C18 ZipTip	0	6	1	6
6	Periyasamy(a)	2015	India	51	Plasma	39/149	Health	Serous epithelial ovarian cancer	MB-HIC C8	33	33	6	116
7	Periyasamy(b)	2015	India	51	Plasma	39/149	Health	Serous epithelial ovarian cancer	MB-HIC C8	34	48	5	101
8	Periyasamy(c)	2015	India	51	Plasma	39/149	Health	Serous epithelial ovarian cancer	MB-HIC C8	36	58	3	91
9	Periyasamy(d)	2015	India	51	Plasma	39/149	Health	Serous epithelial ovarian cancer	MB-WCX	30	32	9	117
10	Periyasamy(e)	2015	India	51	Plasma	39/149	Health	Serous epithelial ovarian cancer	MB-WCX	35	45	4	104
11	Periyasamy(f)	2015	India	51	Plasma	39/149	Health	Serous epithelial ovarian cancer	MB-WCX	27	38	12	111
12	Periyasamy(g)	2015	India	51	Plasma	24/124	Health	Serous epithelial ovarian cancer	IMAC-Cu MBs	19	25	5	99
13	Periyasamy(h)	2015	India	51	Plasma	24/124	Health	Serous epithelial ovarian cancer	IMAC-Cu MBs	21	50	3	74
14	Periyasamy(i)	2015	India	51	Plasma	24/124	Health	Serous epithelial ovarian cancer	IMAC-Cu MBs	19	46	5	78
15	John F. (a)	2010	UK	61.8	Serum	39/22	Benign	OC	C18 ZipTip	24	6	15	16
16	John F. (b)	2010	UK	61.8	Serum	39/66	Health	OC	C18 ZipTip	18	7	21	59
17	Lee, J. H.	2016	South Korea	55.5	Serum	29/24	Benign	OC	NA	26	0	3	24
18	Ali Tiss	2010	UK	NA	Serum	23/29	Benign	OC of CA125 > 30 U/mL	C18 ZipTip	16	4	7	25

*Abbreviations*: *EOC* Epithelial ovarian cancer, *OC* Ovarian cancer, *MB-WCX* Magnetic beads based weak cation exchange, *MB-HIC C8* Hydrophobic interaction chromatography magnetic beads C8, *IMAC-Cu MBs Cu*-immobilized metal affinity chromatography magnetic beads, *NA* Not available, *TP* True positive, *FP* False positive, *FN* False negative, *TN* True negative

### Quality assessment

**Fig. 2:**
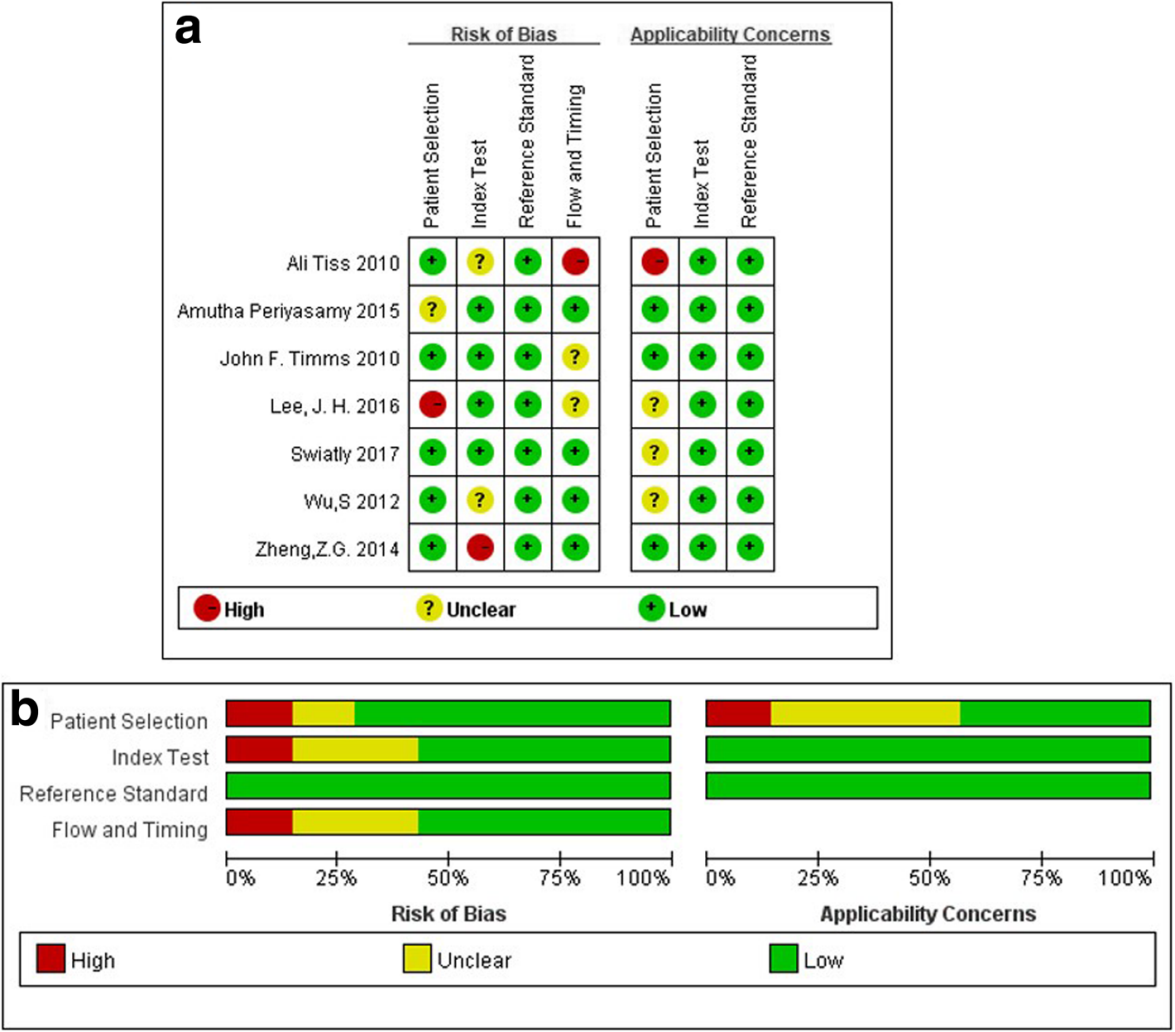
Methodological quality graph (**a**) and summary (**b**)

The quality assessment of these studies was used by Review Manager 5.3 software. Methodological quality analysis of the included studies showed that these articles were at low risk of bias and applicability concerns in total. Although most of the included studies were case-control studies, in the patient selection domain, they enrolled patients consecutively or random samples. Moreover, they avoided inappropriate exclusions. The index tests were performed after the results of the reference standard, otherwise, conducted in double-blind. Some studies’ thresholds were not preset which may affect the interpretation of the results. The risk of the flow and timing domain were at a low level. The summary of the quality assessment was listed in Fig. 2.

### Meta-analysis

**Fig. 3:**
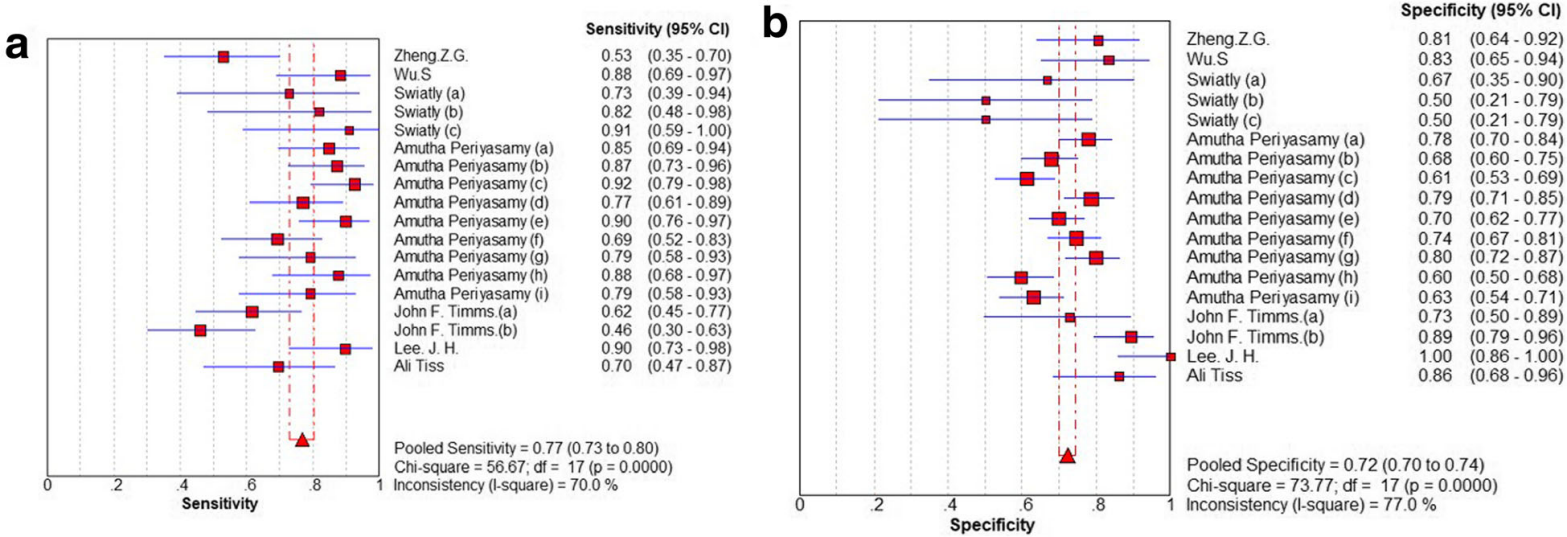
Sensitivity (**a**) and Specificity (**b**) of the diagnostic performance of MALDI-TOF-MS for ovarian cancer

**Fig. 4:**
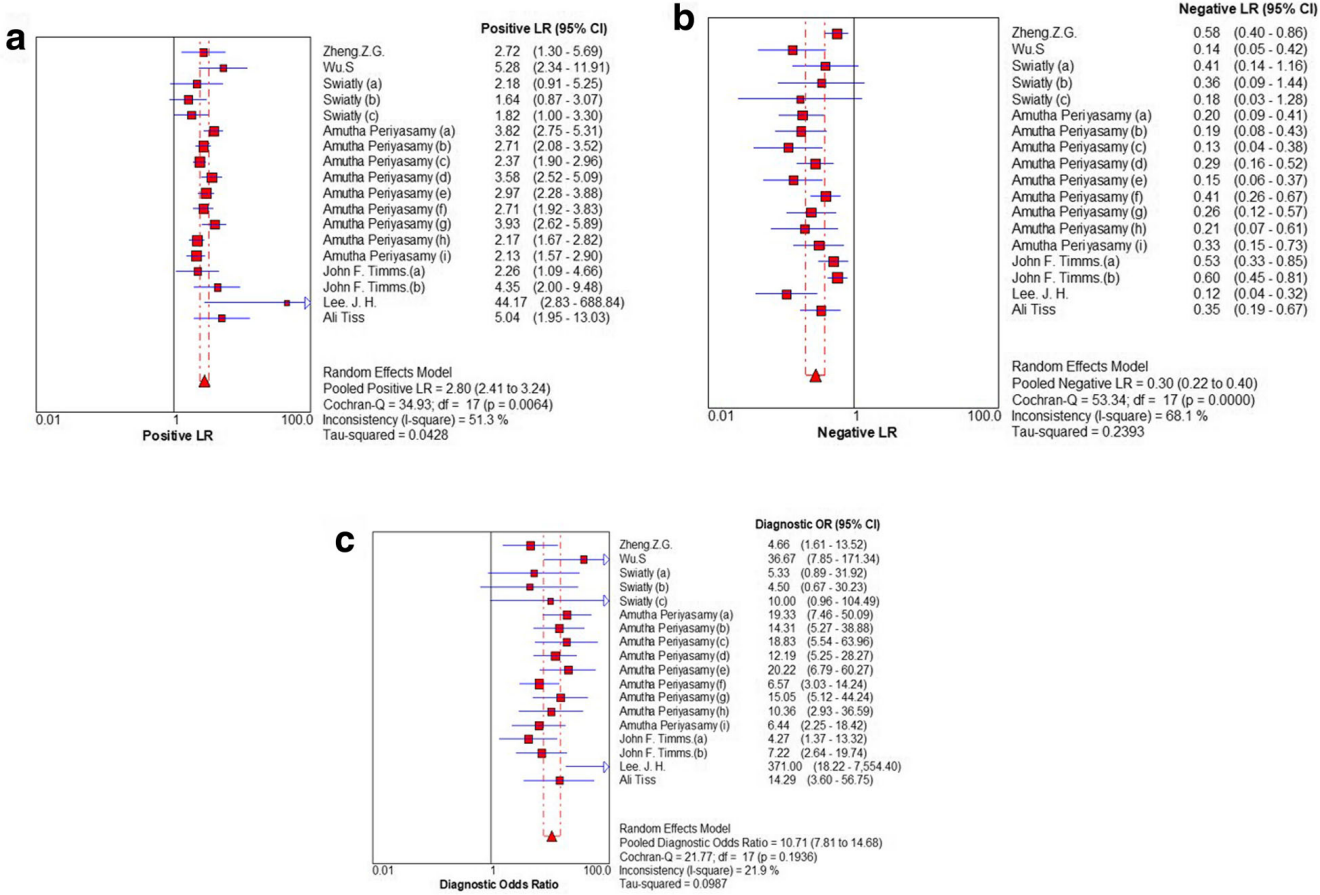
PLR (**a**), NLR (**b**) and DOR (**c**) of the diagnostic performance of MALDI-TOF-MS for ovarian cancer

**Fig. 5:**
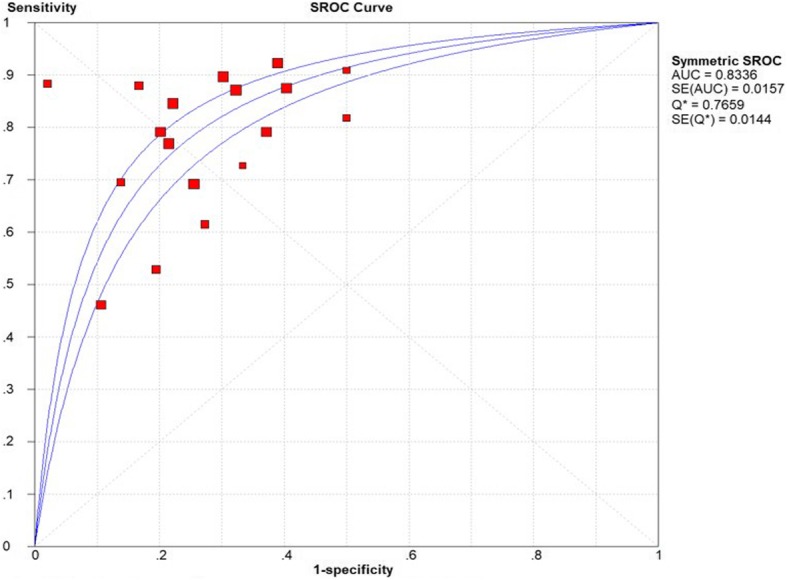
SROC of the diagnostic performance of MALDI-TOF-MS for ovarian cancer

The Spearman correlation coefficient is 0.403 (*P* = 0.097), and the shape of ROC show no typical shoulder arm distribution, the result showed that no threshold effect. Using Meta-Disc1.4 software, we conducted the forest plot of sensitivity and specificity. The *I*^*2*^ of those parameters were more than 50%, it indicated significant heterogeneity among the meta-analysis. Therefore, we pooled the combined data by the random-effects model. Summary estimates of the diagnostic parameters were as follows: sensitivity, 0.77 (95% CI: 0.73–0.80); specificity, 0.72 (95% CI: 0.70–0.74) (Fig. 3), PLR, 2.80 (95% CI: 2.41–3.24); NLR, 0.30 (95% CI: 0.22–0.40) and DOR, 10.71 (95% CI: 7.81–14.68) (Fig. 4). And the AUC was 0.8336 (Fig. 5). It demonstrated that the diagnostic performance of MALDI-TOF-MS for ovarian cancer was a good level of overall accuracy.

### Meta-regression analysis and publication bias

To explore the source of the heterogeneity, we conducted meta-regression analysis based on five variables: region (Asia and others), control (health and others), sample (> 100 and < 100), screening methods (MB-WCX and others) and quality (high and low). Table 2 showed that no source of heterogeneity was observed.

**Table 2:** Meta-regression analysis of the included studies

Covariates	type	number (*n*=)	Co-eff	standard error	*P*-value	RDOR 95%CI
region	Asia	12	0.465	0.365	0.2224	1.59(0.73–3.47)
	others	6				
control	heathy	10	0.211	0.3274	0.5291	1.23 (0.61–2.48)
	others	8				
method	MB-WCX	5	−0.012	0.288	0.9664	0.99 (0.53–1.82)
	others	13				
quality	high	14	−0.487	0.426	0.2708	0.61(0.25–1.52)
	low	4				
sample	> 100	10	0.231	0.3564	0.5275	1.26 (0.59–2.69)
	≤100	8				

*Abbreviations*: *MB-WCX* Magnetic beads based weak cation exchange

**Fig. 6:**
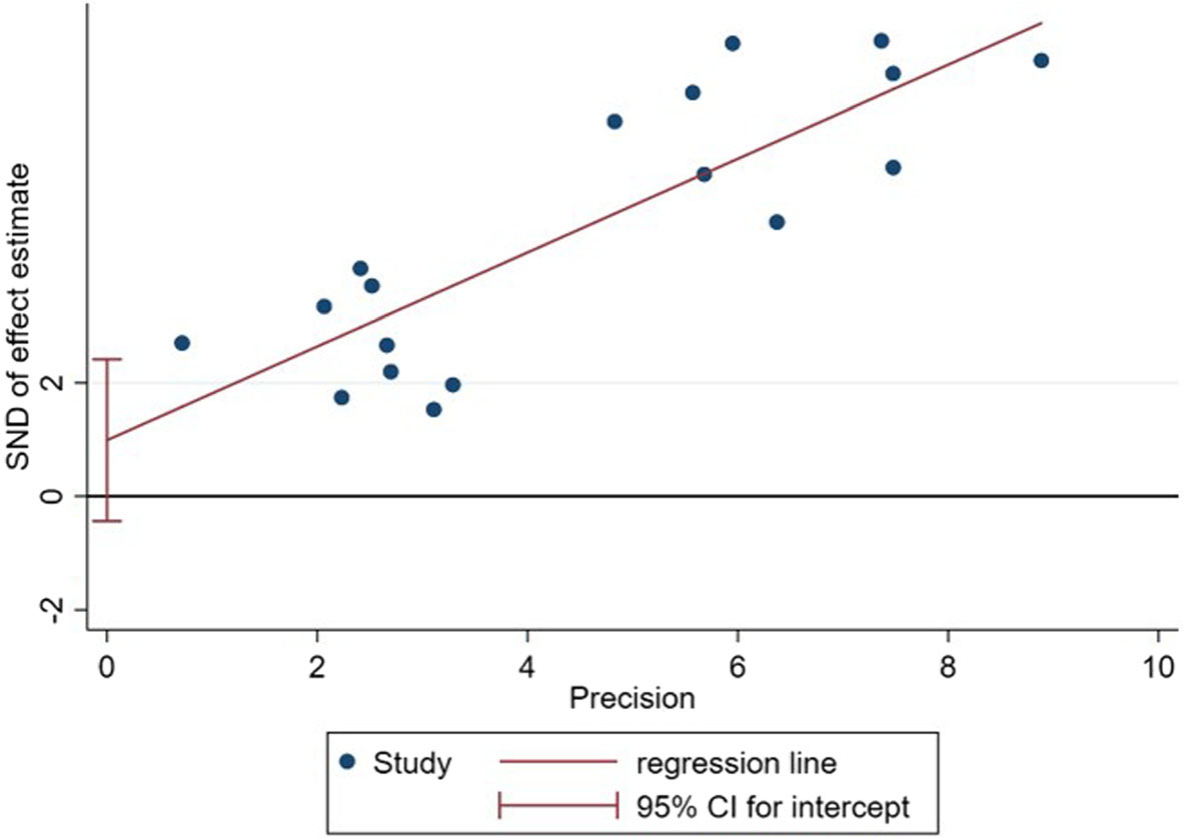
The Egger’s test of the included studies in the meta-analysis

Considered of the number of included articles, the assessment of publication bias was performed by Egger’s test. The results show no significant publication bias in this meta-analysis (*P* = 0.162) (Fig. 6). The results and discussion may be presented separately, or in one combined section, and may optionally be divided into headed subsections.

## Discussion

Nowadays, clinical routine early screening methods involve transvaginal ultrasound examinations (TVUs) and serum CA125 in ovarian cancer. Only 30–40% positive rate still limits the clinical effect [22, 23]. Hence, more efforts are needed to enhance the diagnosis accuracy. Several studies have applied MALDI-TOF-MS to the early diagnosis of ovarian cancer [24–26]. MALDI-TOF-MS is a new type of soft ionization biological protein spectrum technology, which has been developed rapidly in recent years. The stable molecular ions are generated by the soft ionization method, which could not break the measured molecules, and mass spectrometry is carried out by the time of flight mass analyser. The molecular mass of the charged molecules is determined based on the time of arrival at the detector, and a specific fingerprint is performed by special software analysis [27, 28]. Compared with the traditional protein detection technology, the advantages of MALDI-TOF-MS are high sensitivity and accuracy, few sample sizes, short testing periods and varied sample types, such as serum, plasma, tissue, and cerebrospinal fluid [29–31]. It has been used to identify the bacterium in clinical, and then its ability to explore the biomarkers have been gradually developed. MALDI-TOF-MS can also detect the chemotherapy drug resistance, recurrence, and metastasis of ovarian cancer [32–34].

The thorough research included seven articles and contained eighteen studies. In this study, we synthesized the overall diagnostic performance of MALDI-TOF-MS for ovarian cancer. The pooled sensitivity and specificity were 77 and 72%, respectively, which indicated that 23% of patients could be missed and the rate of misdiagnosis was 28%. Compared with CA125 and HE4, the sensitivity and specificity showed moderate performance. The result suggested that multi-marker diagnostic strategies can be used to improve the efficiency. The positive likelihood ratios > 10 and negative likelihood ratios < 0.1 are considered as a significant indicator to judge a diagnosis [35]. In the present meta-analysis, the pooled results that PLR and NLR were 2.80 and 0.30 suggest that the ability of MALDI-TOF-MS may not strong enough to discriminate the ovarian cancer patients from the controls. Lee et al. showed that the best PLR and NLR was respectively 44.17 and 0.13. The reason why the result showed the good performance is that low-mass-ion (LMI) profiling was constructed to screen for OC with the removal of high-abundant peptide and protein [20]. Therefore, the high-abundant protein in serum or plasma should be separated to improve the discovery of meaningful markers when MALDI-TOF-MS is used to differentiate the ovarian cancer. The diagnostic odds ratio [36], a single indicator that shows the degree of association between diagnostic tests and diseases, was 10.71, indicates that MALDI-TOF-MS may be used as an auxiliary rather than independent detection method. The SROC AUC was 0.8336. These results indicated that this technology shows a good ability for diagnosing ovarian cancer and should be used in combination with other markers to improve the accuracy for early diagnosis.

In fact, MALDI-TOF-MS is also used to investigate the abnormal changes of N-glycan modifications on proteins of ovarian cancer. Biskup et al. reported that GLYCOV (a change in serum polysaccharide group in advanced epithelial tumors) can distinguish advanced epithelial ovarian cancer from benign tumors and early epithelial ovarian cancer in more sensitive and specific compared with CA125 [37]. In addition, serum peptide information with CA125 can improve the detectable time for early diagnosis of ovarian cancer [25]. In 2016, OVA2, a second-generation multivariate index FDA-approved assay for ovarian cancer monitor, has already applied in clinical screening for determining the OC risk by clinical information [38]. Ivanova et al. reported that it is necessary to identify the structure of peptide between the patients and other diseases for differential diagnoses [39]. In order to determine the detection threshold better and improve the diagnostic performance, Swiatly et al. found that iTRAQ (isobaric Tags for Relative and Absolute Quantification) coupled with MALDI-TOF-MS was as an appropriate method for identifying different protein expression in serum to improve the ability to discriminate benign tumors from malignant tumors [40].

According to the involved studies, Tiss et al. reported that CA125 showed the good performance in the diagnosis of invasive ovarian cancer and MS profiling have limitation as a diagnostic tool in 2010 [21]. However, with the continuous maturity and improvement of mass spectrometry technology, MS has showed its specific ability in the diagnosis of cancer. Periyasamy et al. reported that the plasma of patients with serous adenocarcinoma can minimize the variability and homogeneity, and the results suggested that MALDI-TOF-MS showed higher sensitivity than specificity in discriminating healthy controls and women with serous adenocarcinoma, the most common type of epithelial ovarian cancer [18]. Swiatly et al. pointed out that the combination of solid phase extraction technology and MALDI-TOF-MS was used to analyse the serum samples. In order to select the best diagnostic model, external validation was implemented with three classification algorithms, including supervised neural network (SNN), genetic algorithm (GA), and quick classifier (QC). In addition, Swiatly et al. showed that SNN was associated with the best differentiating capabilities and satisfactory [17]. Therefore, for the OC patients, the method choosing the solid phase extraction technology and the algorithm choosing SNN may improve the values of sensitivity and specificity when MALDI-TOF-MS are used to diagnose the ovarian cancer.

In this study, we cannot find the source of heterogeneity from five variables. There is no significant publication bias in the meta-analysis. There are only seven articles in this study, and three of them contain more than one study. In particular, one included article contains nine studies, using different statistical algorithms and screening methods to obtain different results [18]. We selected studies according to the inclusion and exclusion criteria as long as the research design is consistent in spite of the type of chips, samples, and algorithms. Thus, any of these may be the source of heterogeneity and affect diagnostic accuracy. As a high precision and extremely sensitive technology, MALDI-TOF-MS has some problems with repeatability. Many factors could affect the stability of the results. From samples collection, experiment operation to data analysis, operators need to control the biased of results within an acceptable range. How to optimize the selection of instrument parameters, standardize of operation produces and reference standard, reduce the difference of various batches is a development path to be a diagnostic tool in the early stages of tumor. Based on the included literature and related reports, if we use MALDI-TOF-MS to diagnose cancer in the early stage, it is necessary to construct a diagnostic model based on statistical algorithms for large sample size firstly. Subsequently, the model should be verified by blind methods. Ultimately, the cut-off value is established in the research results. There are some limitations about this study. The number of included literatures is small, and some of those are included in more than one study, which leads to the loss of diversity of results. Moreover, it is essential that large prospective studies should be determined in order to ensure the diagnostic performance of MALDI-TOF-MS in clinical application. Therefore, higher quality trails with large samples and longer following-up are proposed. We believe that MALDI-TOF-MS will show great application prospect and be a powerful tool for early diagnosis of cancer in the future.

## Conclusions

In conclusion, MALDI-TOF-MS shows a good ability for diagnosing ovarian cancer. However, further evaluation and optimization of standardized procedures are necessary for complete relying on MALDI-TOF-MS to diagnose ovarian cancer.

## Data Availability

The datasets used and/or analysed during the current study are available from the corresponding author on reasonable request.

## References

[1] Ferlay J, Soerjomataram I, Dikshit R (2015). Cancer incidence and mortality worldwide: sources, methods and major patterns in GLOBOCAN 2012. Int J Cancer.

[2] Doherty JA, Peres LC, Wang C, Way GP, Greene CS, Schildkraut JM (2017). Challenges and opportunities in studying the epidemiology of ovarian Cancer subtypes. Curr Epidemiol Rep.

[3] Yurkovetsky Z, Skates S, Lomakin A (2010). Development of a multimarker assay for early detection of ovarian cancer. J Clin Oncol.

[4] Su Z, Graybill WS, Zhu Y (2013). Detection and monitoring of ovarian cancer. Clin Chim Acta.

[5] Kondalsamy-Chennakesavan S, Hackethal A, Bowtell D, Obermair A (2013). Differentiating stage 1 epithelial ovarian cancer from benign ovarian tumours using a combination of tumour markers HE4, CA125, and CEA and patient’s age. Gynecol Oncol.

[6] Karas M, Hillenkamp F (1988). Laser desorption ionization of proteins with molecular masses exceeding 10,000 daltons. Anal Chem.

[7] Menzel C, Dreisewerd K, Berkenkamp S, Hillenkamp F (2002). The role of the laser pulse duration in infrared matrix-assisted laser desorption/ionization mass spectrometry. J Am Soc Mass Spectrom.

[8] Jaskolla TW, Karas M (2011). Compelling evidence for lucky survivor and gas phase protonation: the unified MALDI analyte protonation mechanism. J Am Soc Mass Spectrom.

[9] Lemaire R, Menguellet SA, Stauber J (2007). Specific MALDI imaging and profiling for biomarker hunting and validation: fragment of the 11S proteasome activator complex, Reg alpha fragment, is a new potential ovary cancer biomarker. J Proteome Res.

[10] Cho YT, Su H, Chiang YY (2017). Fine Needle Aspiration Combined With Matrix-assisted Laser Desorption Ionization Time-of-Flight/Mass Spectrometry to Characterize Lipid Biomarkers for Diagnosing Accuracy of Breast Cancer. Clin Breast Cancer.

[11] Yu J, Zhai X, Li X (2017). Identification of MST1 as a potential early detection biomarker for colorectal cancer through a proteomic approach. Sci Rep.

[12] Padoan A, Basso D, Zambon CF (2018). MALDI-TOF peptidomic analysis of serum and post-prostatic massage urine specimens to identify prostate cancer biomarkers. Clin Proteomics.

[13] Whiting PF, Rutjes AW, Westwood ME (2011). QUADAS-2: a revised tool for the quality assessment of diagnostic accuracy studies. Ann Intern Med.

[14] Herrmann D, Sinnett P, Holmes J, Khan S, Koller C, Vassar M (2017). Statistical controversies in clinical research: publication bias evaluations are not routinely conducted in clinical oncology systematic reviews. Ann Oncol.

[15] Zheng ZG, Xia T, Mou HZ, Jiang ZM, Zhou JQ (2014). Serum protein profiling application value in high-grade-serous ovarian cancer diagnosis. Chinese J Cancer Prev Treatment.

[16] Wu S, Xu K, Chen G, Zhang J, Liu Z, Xie X (2012). Identification of serum biomarkers for ovarian cancer using MALDI-TOF-MS combined with magnetic beads. Int J Clin Oncol.

[17] Swiatly A, Horala A, Hajduk J, Matysiak J, Nowak-Markwitz E, Kokot ZJ (2017). MALDI-TOF-MS analysis in discovery and identification of serum proteomic patterns of ovarian cancer. BMC Cancer.

[18] Periyasamy A, Gopisetty G, Veluswami S, Joyimallaya Subramanium M, Thangarajan R (2015). Identification of candidate biomarker mass (m/z) ranges in serous ovarian adenocarcinoma using matrix-assisted laser desorption/ionization time-of-flight mass spectrometry profiling. Biomarkers..

[19] Timms JF, Cramer R, Camuzeaux S (2010). Peptides generated ex vivo from serum proteins by tumor-specific exopeptidases are not useful biomarkers in ovarian Cancer. Clin Chem.

[20] Lee JH, Yoo BC, Kim YH (2016). Low-mass-ion discriminant equation (LOME) for ovarian cancer screening. BioData Min.

[21] Tiss A, Timms JF, Smith C (2010). Highly accurate detection of ovarian cancer using CA125 but limited improvement with serum matrix-assisted laser desorption/ionization time-of-flight mass spectrometry profiling. Int J Gynecol Cancer.

[22] Bourne TH, Campbell S, Reynolds KM (1993). Screening for early familial ovarian cancer with transvaginal ultrasonography and colour blood flow imaging. BMJ..

[23] Carlson KJ, Skates SJ, Singer DE (1994). Screening for ovarian cancer. Ann Intern Med.

[24] Lee JH, Kim YH, Kim KH (2018). Profiling of serum metabolites using MALDI-TOF and triple-TOF mass spectrometry to develop a screen for ovarian Cancer. Cancer Res Treat.

[25] Timms JF, Menon U, Devetyarov D (2011). Early detection of ovarian cancer in samples pre-diagnosis using CA125 and MALDI-MS peaks. Cancer Genomics Proteomics.

[26] Zhao QCHT, Duan W, Wu YM, Qian XH, Deng XH (2008). Analysis of serum biomarkers of ovarian epithelial cancers based on 2-DE DIGE and MALDI TOF/TOF. Zhonghua Zhong Liu Za Zhi.

[27] Villanueva J, Philip J, Entenberg D (2004). Serum peptide profiling by magnetic particle-assisted, automated sample processing and MALDI-TOF mass spectrometry. Anal Chem.

[28] Lopez MF, Mikulskis A, Kuzdzal S (2007). A novel, high-throughput workflow for discovery and identification of serum carrier protein-bound peptide biomarker candidates in ovarian cancer samples. Clin Chem.

[29] Biskup K, Braicu EI, Sehouli J, Tauber R, Blanchard V (2017). The ascites N-glycome of epithelial ovarian cancer patients. J Proteome.

[30] Meding S, Martin K, Gustafsson OJ (2013). Tryptic peptide reference data sets for MALDI imaging mass spectrometry on formalin-fixed ovarian cancer tissues. J Proteome Res.

[31] Segawa S, Sawai S, Murata S (2014). Direct application of MALDI-TOF mass spectrometry to cerebrospinal fluid for rapid pathogen identification in a patient with bacterial meningitis. Clin Chim Acta.

[32] Huang H, Li Y, Liu J (2012). Screening and identification of biomarkers in ascites related to intrinsic chemoresistance of serous epithelial ovarian cancers. PLoS One.

[33] Wang Y, Yu JJ, Zhu T (2012). Analysis of diferentially expressed protein from primary and recurrent ovarian cancer serum. Asian Pac J Trop Med.

[34] Zhang X, Wang Y, Qian Y (2014). Discovery of specific metastasis-related N-glycan alterations in epithelial ovarian cancer based on quantitative glycomics. PLoS One.

[35] Deeks JJ, Altman DG (2004). Diagnostic tests 4: likelihood ratios. BMJ..

[36] Glas AS, Lijmer JG, Prins MH, Bonsel GJ, Bossuyt PM (2003). The diagnostic odds ratio: a single indicator of test performance. J Clin Epidemiol.

[37] Biskup K, Braicu EI, Sehouli J, Tauber R, Blanchard V (2014). The serum glycome to discriminate between early-stage epithelial ovarian cancer and benign ovarian diseases. Dis Markers.

[38] Coleman RL, Herzog TJ, Chan DW (2016). Validation of a second-generation multivariate index assay for malignancy risk of adnexal masses. Am J Obstet Gynecol.

[39] Ivanova OM, Ziganshin RH, Arapidi GP (2016). Scope and limitations of MALDI-TOF MS blood serum peptide profiling in cancer diagnostics. Russian J Bioorganic Chem.

[40] Swiatly Agata, Horala Agnieszka, Matysiak Jan, Hajduk Joanna, Nowak-Markwitz Ewa, Kokot Zenon (2018). Understanding Ovarian Cancer: iTRAQ-Based Proteomics for Biomarker Discovery. International Journal of Molecular Sciences.

